# Self-compensation in arsenic doping of CdTe

**DOI:** 10.1038/s41598-017-04719-0

**Published:** 2017-07-04

**Authors:** Tursun Ablekim, Santosh K. Swain, Wan-Jian Yin, Katherine Zaunbrecher, James Burst, Teresa M. Barnes, Darius Kuciauskas, Su-Huai Wei, Kelvin G. Lynn

**Affiliations:** 1 National Renewable Energy Laboratory, Golden, CO 80401 USA; 2 Center for Materials Research, School of Mechanical and Materials Engineering, Washington State University, Pullman, WA 99164-2711 USA; 3 College of Physics, Optoelectronics and Energy & Collaborative Innovation Center of Suzhou Nano Science and Technology, Soochow University, Suzhou, 215006 China; 4 Beijing Computational Science Research Center, Beijing, 100094 China

## Abstract

Efficient p-type doping in CdTe has remained a critical challenge for decades, limiting the performance of CdTe-based semiconductor devices. Arsenic is a promising p-type dopant; however, reproducible doping with high concentration is difficult and carrier lifetime is low. We systematically studied defect structures in As-doped CdTe using high-purity single crystal wafers to investigate the mechanisms that limit p-type doping. Two As-doped CdTe with varying acceptor density and two undoped CdTe were grown in Cd-rich and Te-rich environments. The defect structures were investigated by thermoelectric-effect spectroscopy (TEES), and first-principles calculations were used for identifying and assigning the experimentally observed defects. Measurements revealed activation of As is very low in both As-doped samples with very short lifetimes indicating strong compensation and the presence of significant carrier trapping defects. Defect studies suggest two acceptors and one donor level were introduced by As doping with activation energies at ~88 meV, ~293 meV and ~377 meV. In particular, the peak shown at ~162 K in the TEES spectra is very prominent in both As-doped samples, indicating a signature of AX-center donors. The AX-centers are believed to be responsible for most of the compensation because of their low formation energy and very prominent peak intensity in TEES spectra.

## Introduction

Cadmium telluride (CdTe) and its alloys such as mercury cadmium telluride (HgCdTe) and cadmium zinc telluride (CdZnTe) are important electronic materials a with wide range of applications including photovoltaics, medical imaging, X-ray and gamma-ray detection. Many CdTe-based semiconductor devices require highly p-type doped material with long minority carrier lifetimes^1, 2^, however, efficient p-type doping in CdTe is challenging^3^ and the mechanism behind it^4^ is not yet well understood. The major limiting factors may include low solubility of dopants where dopants could become clusters forming secondary phases and self-compensation^5–7^. The exact mechanism of self-compensation is still unclear. Fundamental understanding of the p-type doping mechanism in CdTe is needed to provide critical insights to improve the p-type doping efficiency^8^.

As a choice for p-type doping, arsenic (As) has been a topic of interest in CdTe, especially in HgCdTe^9, 10^ for infrared technologies using wide range of thin film deposition techniques. Using As for p-type doping is also attractive for photovoltaics^11^. Recently, phosphorus (P) emerged^12, 13^ as viable alternative p-type dopant in CdTe solar cells, but As seems to be a more appropriate choice because it is believed to be safer in high-volume manufacturing compared to P and it also has higher solubility. Experimental work has identified As as shallow acceptor^14^ (<100 meV), but activation of As has been difficult^15, 16^. High temperature post-growth annealing activation steps with optimized stoichiometry control are generally required to produce high concentrations of acceptors^17, 18^. Although significant progress has been made, the success of As-doping is still limited because of the short carrier lifetime^16^. Theoretical predictions suggest^19, 20^ As-doping in CdTe could be limited by the formation of self-compensating AX-center defects. The AX-center is an acceptor-induced defect that acts as a donor to compensate the acceptor itself. AX-centers are formed when substitutional acceptors undergo a transition from shallow acceptor states to deep donor states with large lattice relaxations in the vicinity of the impurities. However, no experimental observation of AX-centers has been reported in CdTe.

Here, we report the indication of the existen﻿ce of AX-centers based on a systematic analysis of defect states in As-doped CdTe that combines well-controlled crystal growth, experimental measurements, and theoretical calculations. CdTe single crystals were grown from melt using the vertical Bridgman technique, where the growth details are described elsewhere^21, 22^. Defects in the samples were investigated by thermoelectric-effect spectroscopy (TEES). First-principles calculations based on hybrid functional methods of As-related defects and defect complexes are used to interpret the experimental data and confirm defect assignments.

## Results

Four types of bulk crystals, summarized in Table 1, were grown for this study to identify the defects exclusively due to As-doping and exclude native defects by comparing the defect signatures with undoped crystals grown under varying stoichiometry. The amount of excess Cd and dopant concentration (*C*_*p*_ in melt) used in each growth are also detailed. The incorporated dopant concentration in as-grown boules (*C*_*p*_ after growth) was determined using glow discharge mass spectrometry (GDMS) (see Supplementary Table S1 for impurity levels of as-grown crystals). The carrier density *N*_*A*_ and resistivity (ρ) were measured by room temperature Hall-effect measurements in the van der Pauw configuration. The activation efficiency of dopants was determined by comparing the (Hall) measured *N*_*A*_ to the dopant concentration measured by GDMS (*C*_*p*_ after growth)^23, 24^. The aggregate bulk minority carrier lifetimes (τ) were determined using two-photon excitation time-resolved photoluminescence (2PE-TRPL)^25, 26^.

**Table 1:** Growth conditions and electrical properties of representative samples. The concentration of dopants (*C*_*p*_) and excess Cd in the melt are the intended concentrations used in the charge for growth. The uncertainty in the excess Cd is ± 2 × 10^18^ cm^−3^. Samples A1, A2, and B2 are p-type.

Sample	Dopant	Growth	Excess Cd (cm^−3^)	*C*_*P*_ (in melt) (cm^−3^)	*C*_*P*_ (after growth) (cm^−3^)	*N*_*A*_ (cm^−3^)	ρ (Ω.cm)	Activation (%)	Lifetime (ns)
A1	As	Cd-rich As-doped	6 × 10^18^	5.5 × 10^17^	1.2 × 10^17^	(3–5) × 10^13^	~800	0.02–0.04	6.5
A2	As	Cd-rich As-doped	6 × 10^18^	1.1 × 10^17^	(5–10) × 10^16^	(1–2) × 10^15^	~20	1–3	~1.4
B1	None	Cd-rich Undoped	5 × 10^18^	None	None	N/A	N/A	N/A	245–300
B2	None	Te-rich Undoped	N/A	None	None	~1 × 10^15^	100–500	N/A	31–236

Samples A1 and A2 are As-doped CdTe crystals grown in Cd overpressure conditions. The A2 growth used a lower amount of As in the melt and identical Cd overpressure. A2 was cooled to room temperature after growth at an average rate of ~52 °C/hr, whereas A1 was cooled at 7 °C/hr. The reason for performing faster cooling on A2 was to observe the effect of cooling rate on dopant solubility and incorporation. Adding extra Cd in the As-doped growths is intended to facilitate substitutional dopant incorporation on the Te site. Samples B1 and B2 are undoped CdTe crystals grown with and without excess Cd vapor pressure, respectively. Due to the higher vapor pressure of Cd over Te, the B2 growth is expected to be Te-rich. The labels “Cd-rich” and “Te-rich” refer to the growth stoichiometry. The amount of added extra Cd is beyond the solubility limit as mentioned in ref. 27 to produce deviation from stoichiometry. Theoretical predictions have suggested controlling growth stoichiometry is key for obtaining long minority carrier lifetime^28^.

**Figure 1:**
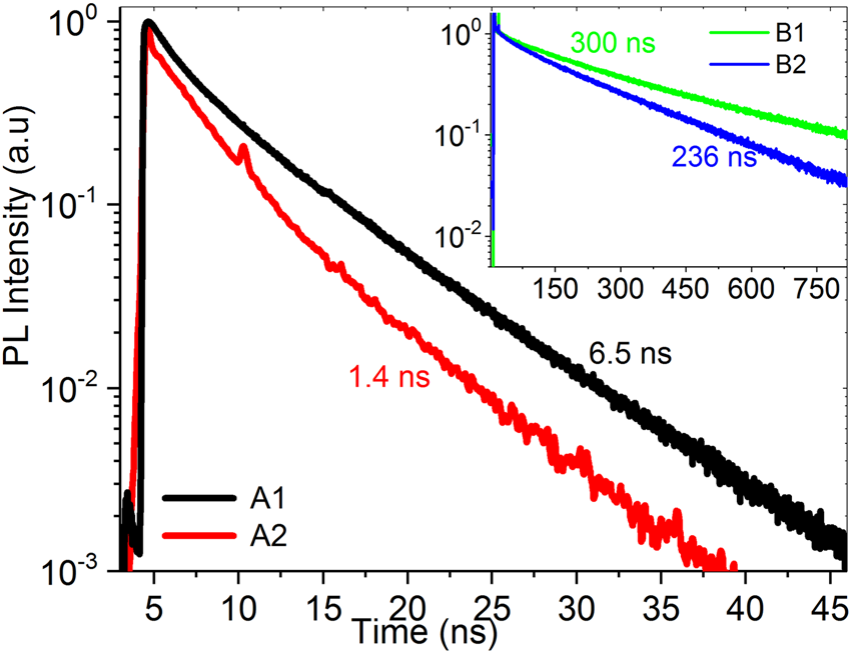
Normalized two-photon excitation TRPL decays for longest lifetimes in samples. Excitation was at 1120 nm and photoluminescence emission was measured at 840 nm. The inset is TRPL decays for samples B1 and B2.

Table 1 shows *N*_*A*_ in A1 is ~10^13^ cm^−3^ range, which is four orders of magnitude lower than the arsenic concentration *C*_*p*_ = ~10^17^ cm^−3^ (measured by GDMS), suggesting 0.02%–0.04% activation. The extremely low activation of dopant indicates the sample is severely compensated. The *N*_*A*_ in A2 is in the low − 10^15^ cm^−3^ range, which is two orders of magnitude higher than in A1. The activation in A2 is around 1%–3%; however, this sample is still highly compensated since ~97% of the As is not contributing to free carriers. For carrier lifetimes in the samples, measured TRPL decays are shown in Fig. 1. The τ in B1 is 245–300 ns. The τ in B2 had more spatial variation, with a range of lifetime values varying from ~30 ns to 236 ns, indicating non-uniformity in the bulk, probably due to Te inclusions or precipitates. The fact that the Cd-rich growth had a longer lifetime than the Te-rich growth agrees with the theory predictions that Te_Cd_ antisite defects are efficient recombination centers characterized by low formation energy^28^. The τ in the As-doped samples A1 and A2 are around 1–6 ns. In contrast, P-doped CdTe materials grown in similar conditions have shown high *N*_*A*_ (10^16^–10^17^ cm^−3^) and high lifetime (55–400 ns) with around 50% activation^12^ suggesting the doping with As is severely limited. The As-doped crystals are characterized by low carrier density, low dopant activation and short lifetime, suggesting strong self-compensation and the presence of significant carrier trapping defects.

The defect states in each type of CdTe crystals were extensively studied by the TEES method. The thermal ionization energy (*E*_*th*_) and trapping cross sections (*σ*_*th*_) were calculated by the variable heating rate (VHR) method. In the VHR technique, the measurement is repeated with gradually increasing heating rates, which shifts the peak positions (*T*_*M*_) to higher temperatures. In perfect testing conditions where the contacts are Ohmic, the sample is highly resistive and hole and electron mobility are similar, the positive and negative TEES currents correspond to hole and electron traps. However, in the case of p-type CdTe, making Ohmic contact to CdTe is challenging. Adding a small amount of Cu before the contact improves the contact quality, but the Cu may diffuse into the sample creating additional defects, which we want to avoid. Also, the hole and electron mobility in CdTe differs by roughly 10 times. In such conditions, carriers have higher chances of being collected from one side than the other. In most TEES measurement on low resistivity (ρ < 10^7^ Ω.cm) CdTe samples, the observed TEES peaks are either all positive or negative. Therefore, the sign of current peaks may not correspond to the trap types.

**Figure 2:**
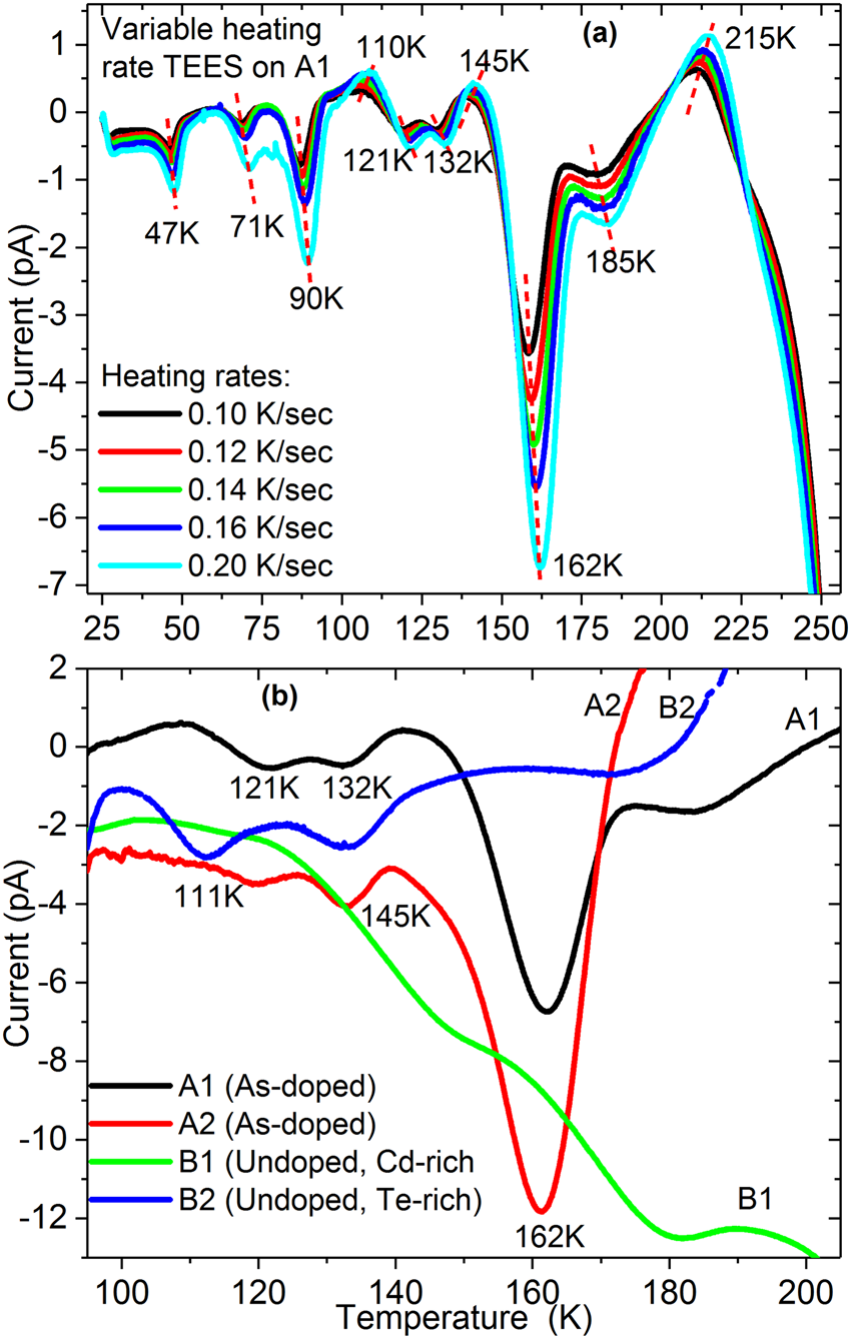
(**a**) Variable heating rate TEES spectrum of sample A1. Peak locations are labeled with respect to 0.2 K/sec heating rate, and (**b**) TEES spectrum comparison of all samples with 0.2 K/sec heating rates.

The TEES spectrum with VHR method on the As-doped sample A1 is presented in Fig. 2(a), in which we observed 10 peaks before current saturation. The most pronounced peak in the spectrum is found to be at ~162 K. Further TEES analysis were performed on other samples A2, B1 and B2 to identify which peaks are induced by arsenic doping and which are related to native point defects. The Fig. 2(b) shows the TEES spectrum of all samples between 100 K and 200 K to track the ~162 K peak for comparison. In different samples or in different testing conditions, a similar peak can move to either side ±5 K.

The defect peaks in each sample were carefully investigated with various system parameters and the confirmed peak positions are summarized in Table 2. Peaks P1, P5 and P8 at 47 K, 121 K and 162 K are only observed in As-doped samples. The remaining seven peaks (P2, P3, P4, P6, P7, P9, and P10) were observed in all samples. These seven peaks in different samples appeared at similar temperatures and the extracted *E*_*th*_ values of these seven peaks are within 10% of each other in all four samples, suggesting these peaks correspond to similar types of defects. We assigned them to native defects independent of extrinsic doping. These native defects in undoped samples are discussed in ref. 29, where the equations and an example of extracting defect parameters are given. In this paper, we only focus on the defects induced by arsenic doping. Clearly, the peaks P1, P5 and P8 were associated with defects induced by As-doping.

**Table 2:** TEES current maxima positions observed in samples (letter P represents peak). “Yes” means the peak is observed, and “No” means the peak is not observed.

Peak	Peak positions (K)	A1	A2	B1	B2
P1	46–48	Yes	Yes	No	No
P2	69–73	Yes	Yes	Yes	Yes
P3	89–93	Yes	Yes	Yes	Yes
P4	110–113	Yes	Yes	Yes	Yes
P5	121	Yes	Yes	No	No
P6	132–135	Yes	Yes	No	Yes
P7	142–147	Yes	Yes	Yes	Yes
P8	162	Yes	Yes	No	No
P9	183–185	Yes	Yes	Yes	Yes
P10	215–219	Yes	Yes	Yes	Yes

**Figure 3:**
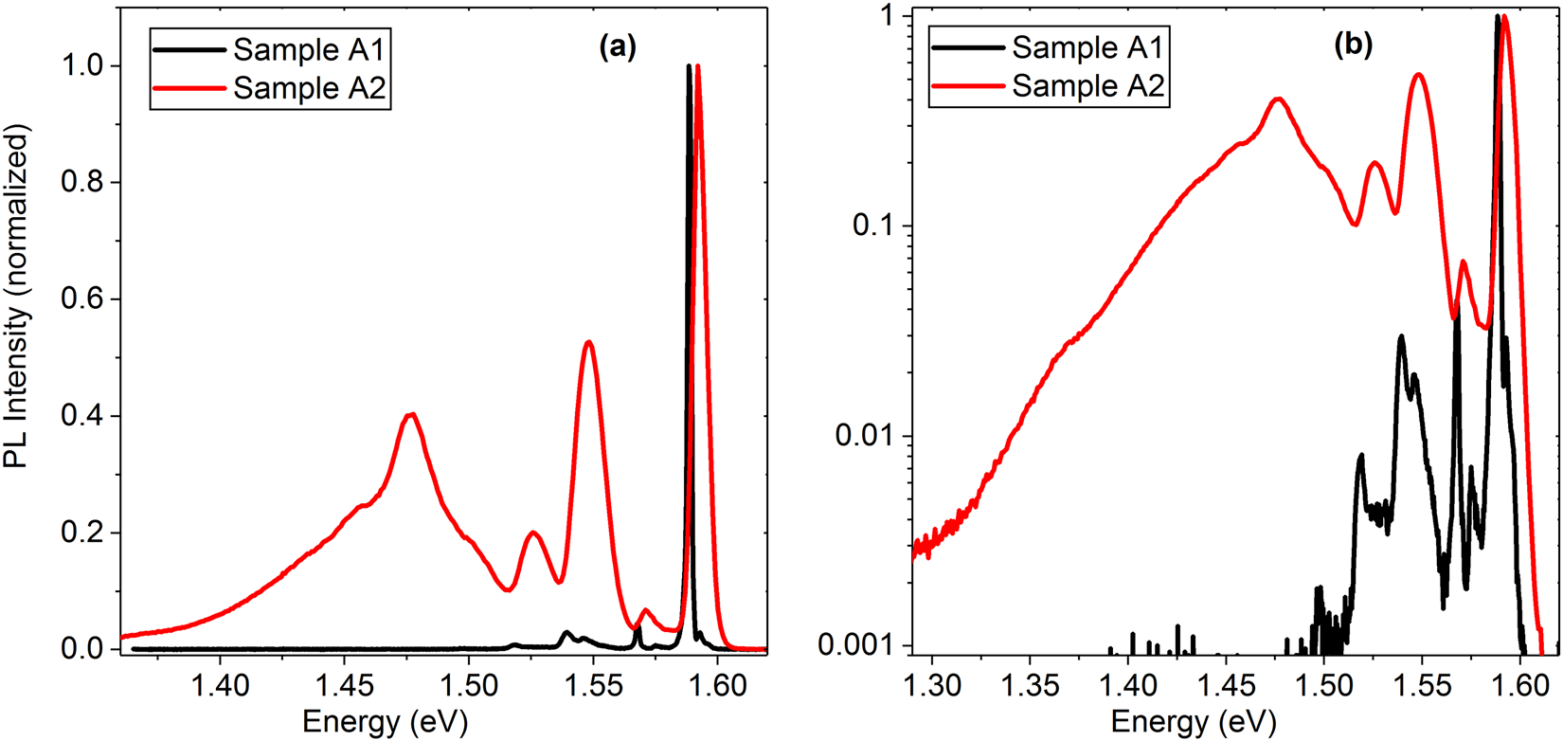
Normalized low temperature (4.25 K) PL emission spectra for samples A1 and A2. Data are plotted in linear (**a**) and log scale (**b**).

For comparison with TEES data, low-temperature photoluminescence (PL) emission spectra for samples A1 and A2 are shown in Fig. 3. The strongest PL emission peaks at 1.589 eV (A1) and 1.592 eV (A2) are attributed to acceptor bound excitons^30^. Exciton emission peak for A1 is narrower (full-width-at-half-maximum 10 and 60 meV for A1 and A2, respectively), which is in agreement with lower doping for this crystal. LO phonon (energy 21 meV) replicas for the exciton emission are at 1.568 eV (A1) and 1.571 eV (A2). PL emission at ≈1.55 eV is attributed to point defects, most likely donor-to-valence band and donor-to-acceptor emission^31^. Broad PL emission at 1.474 eV for A2 is attributed to the Y band (excitons bound to Te glide dislocations) and its phonon replicas^32^. No such emission is observed for A1, which could be related to different surface preparation. PL emission peaks at 1.519 eV (A1; LO phonon replica at 1.498 eV) and 1.526 eV (A2) could be related to As_Te_ dopant^33, 34^. Dopant activation energy from the low-temperature PL emission data (0.08–0.09 eV) is in good agreement with the TEES results and the first-principles analysis (see below).

We note that PL emission spectroscopy probes near-surface properties (1/α_632.8nm_ = 0.2 μm, where α_632.8nm_ is the absorption coefficient at the excitation wavelength). Because near-surface charge carrier lifetimes are much shorter than the carrier lifetimes in the bulk^25^, bulk characteristics studied by TEES and 2PE-TRPL could depend on different defects than surface properties analyzed by PL emission spectroscopy. PL spectroscopy is also less sensitive to emission from the deep defect states, which are more efficiently quenched by multi-phonon emission^35^.

**Figure 4:**
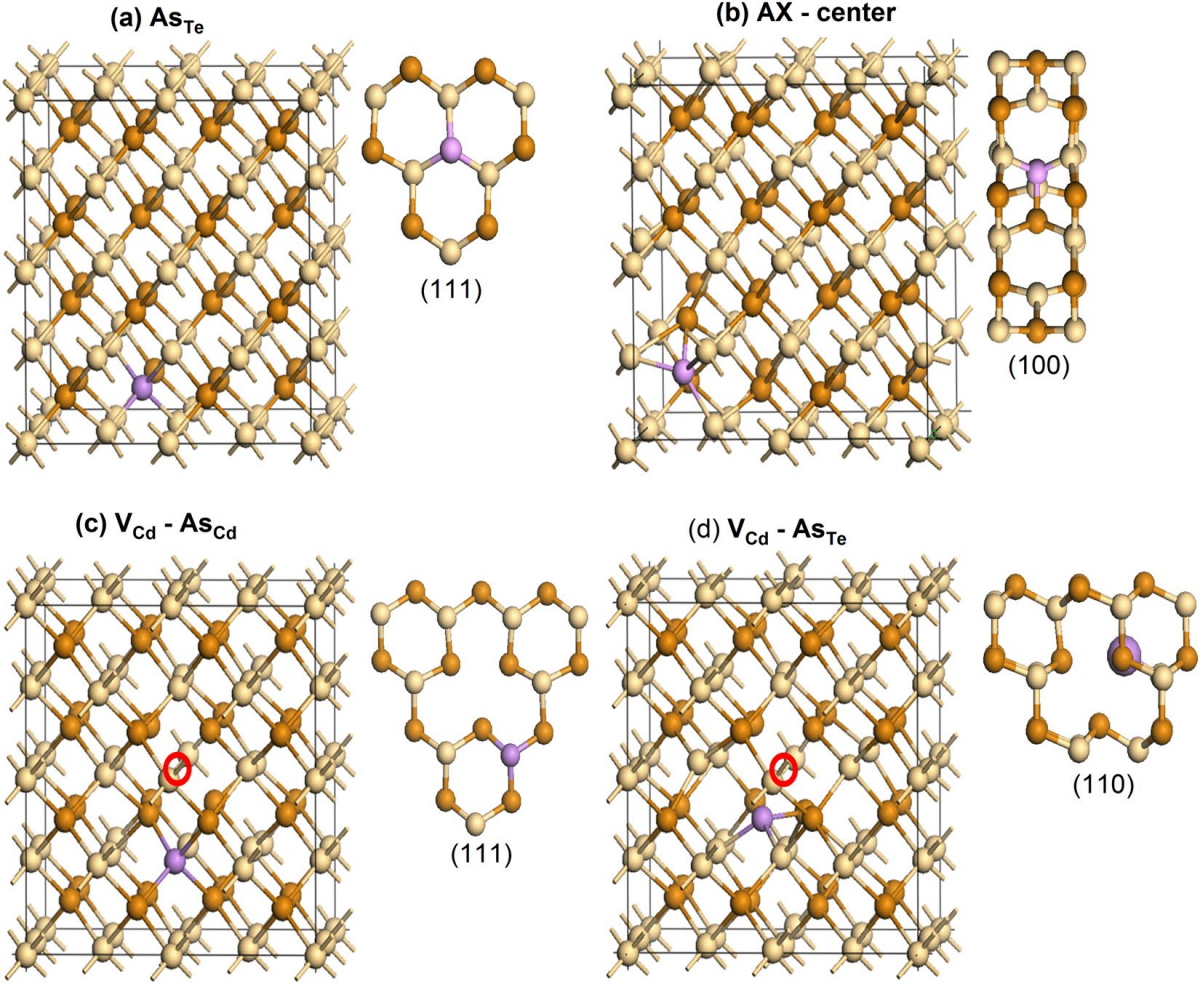
Atomic structures of (**a**) As_Te_, (**b**) AX-center, (**c**) V_Cd_ − As_Cd_, and (**d**) V_Cd_ − As_Te_.

**Figure 5:**
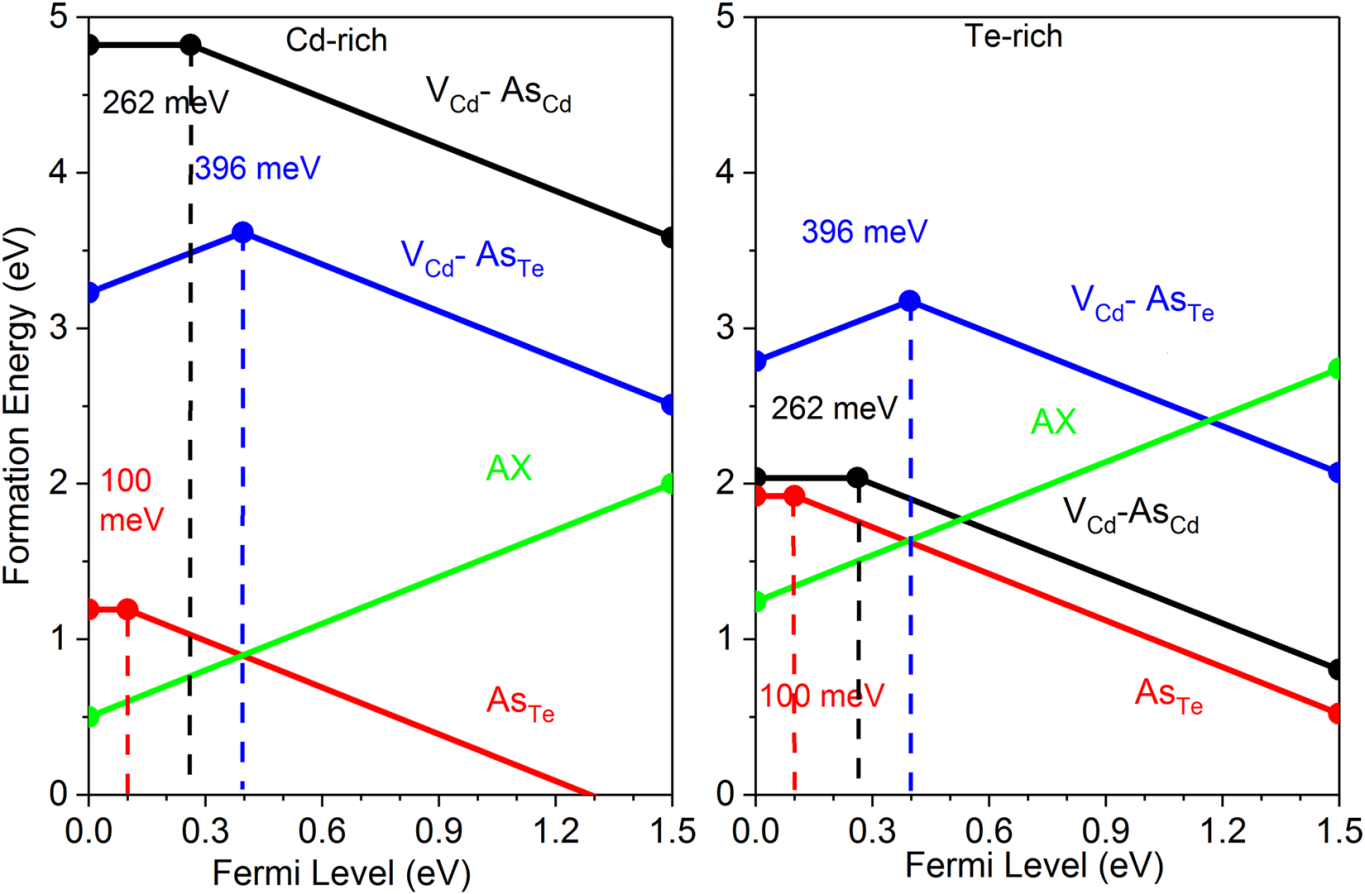
Formation energies of As_Te_, AX-center, V_Cd_ − As_Cd_ and V_Cd_ − As_Te_ in (**a**) Cd-rich and (**b**) Te-rich conditions.

Next, we analyze defect properties form the first-principles calculations. Compensating defects in CdTe:As could be formed by two mechanisms: i) doping could create donors by forming AX-centers, and ii) As can go to a Cd site or form a defect complex with Cd vacancies (V_Cd_) such as (V_Cd_ − As_Cd_) and/or (V_Cd_ − As_Te_). To interpret the experimental observations, we calculated the formation energy and transition energies of most likely defects As_Te_, the AX-center, and two kinds of defect complexes (V_Cd_ − As_Cd_) and (V_Cd_ − As_Te_) with first-principle calculations using VASP code^36^ with hybrid functional methods (PBE0)^37^. The atomic structures of these defect complexes are shown in Fig. 4 and the calculated formation energies are shown in Fig. 5.

The experimental *E*_*th*_ and *σ*_*th*_ values extracted by the VHR method from the P1 peak at ~47 K are 88 ± 3 meV and (2.9 ± 0.7) × 10^−17^ cm^2^, respectively. The literature reports the activation energy of an arsenic atom occupying a Te site as an acceptor is 90–92 meV in several reports^33, 34, 38, 39^. Our theoretical calculations showed the (0/−) transition level of As_Te_ is 100 meV, which matches (88 ± 3 meV) quite well. Therefore, the P1 peak is assigned to accepter state of As substituting Te site, ${{\rm{As}}}_{{\rm{Te}}}^{-/0}$. This relatively shallow level is responsible for *p*-type conductivity of As-doped CdTe.

For the P5 peak at ~121 K, the *E*_*th*_ = 293 ± 12 meV and *σ*_*th*_ = (2.3 ± 1.4) × 10^−15^ cm^2^. This peak was observed in both A1 and A2, but the peak area is much lower than the 162 K peak, i.e., it has a lower trap concentration. Its trapping cross section is on the order of 10^−15^ cm^2^, suggesting significant carrier trapping if present in the sample with significant concentration. We propose it could be associated with (V_Cd_ − As_Cd_) acting as acceptor. According to calculated formation energy, it should have low concentrations in the Cd-rich condition but could dominate in Te-rich conditions. Although the calculated formation energy is high, it is still possible for the As_Cd_ donor to combine with V_Cd_ acceptor and form a (V_Cd_ − As_Cd_) defect complex in the Cd-rich growth case. Our calculated (0/−) transition level for the defect complex is 262 meV, which agrees with the experimental data.

The peak P8 at ~162 K is very prominent in the TEES spectra of both samples A1 and A2, indicating significant and dominant trap density. This peak has not been previously observed in any type of CdTe/CdZnTe samples in our TEES measurements. The *E*_*th*_ and *σ*_*th*_ values for this peak are 377 ± 12 meV and (1.9 ± 0.4) × 10^−15^ cm^2^, respectively. Since the CdTe: As is highly compensated, we hypothesize this peak represents the primary compensating donor defect. Previous theoretical work has predicted p-type doping efficiency in CdTe could be limited by self-compensating defects, including the AX-center. When an AX-center is formed, the As atom will move toward its neighboring Te atom and form an As-Te bond by breaking their two bonds with Cd^40^. Earlier theoretical studies^5^ on AX-Centers have suggested AX-centers in CdTe are metastable and not a limiting factor in p-type doping. However, more recently, Biswas *et al*. predicted^41^ significantly enhanced stability of AX-centers in II–VI semiconductors. Furthermore, Yang *et al*. showed that AX-centers in CdTe are easy to form and should be present with significant concentrations^40^. We have found both AX-center and (V_Cd_ − As_Te_) could contribute to the P8 peak due to their similar transition energies. Our calculated (+/−) transition level for AX^+^ center to As_Te_^−^ is 394 meV and for (V_Cd_ − As_Te_), the (+/−) transition is 396 meV, both of which are consistent with the experimentally determined 377 ± 12 meV activation energy. However, the formation energies of AX-centers in both Cd-rich and Te-rich conditions are much lower than (V_Cd_ − As_Te_). Therefore, P8 is dominated by the AX-centers. Yang *et al*. also proposed the formation of AX-centers can be suppressed by rapid cooling to sustain the hole density and overcome the Fermi level pinning^40^. Although it is not as rapid as quenching, the effect of cooling rate is two orders of magnitude higher *N*_*A*_ in sample A2 than A1, where the cooling rate of ~52 °C/hr in A2 is much faster than 7 °C/hr in A1.

IR microscopy has shown there are also a significant number of clusters in the form of secondary phases (SPs) present in samples A1 and A2, which could have potentially contained some dopants in them and contributed to the low level of dopant incorporation in the lattice. Our initial analysis suggests SPs are related to the dopants, such as compounds of As. Future studies will provide more insights into the origin of this clustering.

In summary, experiments and theoretical calculations of the defect states in As-doped high-purity CdTe indicate that As-doping introduced two acceptors and one compensating donor as outlined in the Table 3. Analysis showed the prominent peak observed at ~162 K in the TEES spectra is most likely related to the AX-centers which can severely limit the p-type doping efficiency. The results contribute towards a better fundamental understanding of defect and dopant chemistry for CdTe. The measurements and theory identify a key barrier to producing highly doped CdTe, and the experiments suggest that changing the cooling rate and growth stoichiometry may help overcome this barrier. The data and analysis provide a basis and guidance for the materials science applications of several electronic and optical technologies. Especially in CdTe photovoltaics (PV) technology, using As-doped CdTe as an absorber in the cell structure has great potential^42^. However, more investigations are needed to achieve material quality requirements for the next generation cell efficiency beyond 22%. As identified in the current study, reducing self-compensation and suppressing the formation of AX-centers will be important steps that could be achieved with methods such as quenching^40^ and careful stoichiometry control.

**Table 3:** Thermal ionization energy (*E*_*th*_), trapping cross sections (***σ***_***th***_) of the defects associated with As-doping observed in TEES spectrum and theoretically calculated transition levels and defect characters.

Peak	T_m_ (K)	*E*_*th*_ by TEES (meV)	*σ*_*th*_ (cm^2^)	E_tran_ by HSE06 (meV)	Characteristics
P1	47	88 ± 3	(2.9 ± 0.7) × 10^−17^	100	As_Te,_ acceptor
P5	121	293 ± 12	(2.3 ± 1.4) × 10^−15^	262	(V_Cd_ − As_Cd_), acceptor
P8	162	377 ± 12	(1.9 ± 0.4) × 10^−15^	396	AX-center and (V_Cd_ − As_Te_) but AX-center is dominant. Both are donors.

## Methods

### Two-photon excitation time-resolved photoluminescence (2PE-TRPL)

The single-crystal samples used for the measurement of the carrier lifetimes in the bulk were approximately 10 × 10 × 1 mm^3^. Sample surfaces were fine polished. The laser system provided 1120-nm excitation using 0.3-ps laser pulses with a 1.1-MHz repetition rate. Several spots, with an analysis volume of approximately 40 μm (lateral) × 120 μm (axial), were measured in each sample and carrier lifetimes variation is reported in Table 1.

### Thermoelectric-effect Spectroscopy (TEES)

For the measurement, CdTe samples with dimensions 10 × 10 × 1 mm^3^ were mechanically polished with alumina paste and lightly etched with Br/methanol solution. Planar gold contacts were sputtered on both faces for electrical contacts. Then the samples were sandwiched between two planar gold contacts and placed on top of a cryostat where they were cooled in the dark down to ~20 K to be illuminated by a sub-band gap LED light source to excite carriers and fill the traps. The peak wavelength of the LED used was 940 nm. Then, samples were heated independently by two resistive heaters from both the top and bottom, which maintained the intended temperature gradient ΔT = 10 K across the sample. As the samples were heated, the charge carriers emanating from the traps produce measurable currents (pA) and form current peaks as a function of temperature, which were measured by a Keithley 6517 A electrometer. The temperature position of the peaks (peak position) is related to the thermal ionization energy (*E*_*th*_) and carrier capture cross section (*σ*_*th*_) of the defect levels. The *E*_*th*_ and *σ*_*th*_ values can be calculated by fitting temperature maxima (*T*_*m*_) in a variable heating rate method (VHR)^43^. The details of theoretical and experimental aspects in TEES can be found in ref. 44 and examples using TEES for extracting *E*_*th*_ and *σ*_*th*_ in CdTe/CdZnTe are available in refs 45, 46.

### PL Emission Spectroscopy

A He-Ne continuous-wave laser with a center wavelength of 632.8 nm was used for excitation. The PL detector was Si CCD with a spectral resolution of 0.3 nm. Low-temperature measurements were performed with a helium-cooled cryostat. The beam diameter at the sample position was 250 µm and the excitation power was 1 mW.

### First-principle calculations

The defect calculation uses the experimental lattice constant (6.48 Å) and (2 × 2 × 2) supercell with 64 atoms. The energy cut-off for basis functions is 400 eV. We used Γ-centered (2 × 2 × 2) *k*-point mesh for Brillouin zone integration. The atomic positions inside the supercell are relaxed until all of the forces on the atoms are less than 0.01 eV/Å. The details of the defect calculation methods used in this work can be found in refs 3, 47. In defect calculations, the computational errors can be attributed to the finite supercell size, incorrect band gap and the used exchange-correlation method. For typical calculation under GGA, the error for transition levels could be up to 0.1 eV^3, 47^. In the present calculation, we used hybrid functional methods to correct the band gap of CdTe to 1.50 eV, compared to the GGA band gap of 0.78 eV. Within an accurate band gap, the errors for transition levels should be much less than previous estimations on GGA calculation.

## Electronic supplementary material

Supplementary Table S1.
