# Supplementary material for: Self-compensation in arsenic doping of CdTe
Source: Sci Rep. 2017 Jul 4;7:4563. doi: 10.1038/s41598-017-04719-0 (PMC5496905; doi:10.1038/s41598-017-04719-0)
Supplement: Supplementary file 1 — Supplementary Table S1. [file 41598_2017_4719_MOESM1_ESM.pdf]

Self-compensation in arsenic doping of CdTe

Tursun Ablekim, Santosh K. Swain, Wan-Jian Yin, Katherine Zaunbrecher, James Burst, Teresa M. Barnes, Darius Kuciauskas, Su-Huai Wei and Kelvin G. Lynn

Supplementary Tables

Supplementary Table S1. Glow-discharge mass spectrometry (GDMS) data analysis for samples used in the study. The units are parts per billion (ppb) atomic. ND represents for "not detected".

Elements	A1	A2	B1	B2
	As-doped	As-doped	Undoped Cd-rich	Undoped Te-rich
Li	19	ND	ND	ND
C	20	330	120	370
N	7	95	30	10
O	35	290	400	240
Na	120	20	130	31
Mg	29	26	20	94
Al	6	10	51	22
Si	ND	13	120	3
P	ND	4	ND	6
S	ND	26	87	29
Cr	ND	ND	100	14
Fe	ND	93	100	180
Ni	ND	ND	ND	61
Cu	ND	8	25	20
Total Impurities	236	915	1183	1080
Without C and O	181	295	663	470
